# Multiaxial Fatigue Behavior of CFRP Thin-Walled Tubes: An Experimental Study with Analysis of the Acoustic Signals

**DOI:** 10.3390/polym17192701

**Published:** 2025-10-07

**Authors:** Szymon Duda, Michał Smolnicki, Paweł Zielonka, Paweł Stabla, Grzegorz Lesiuk

**Affiliations:** Faculty of Mechanical Engineering, Wroclaw University of Science and Technology, 50-370 Wroclaw, Poland; michal.smolnicki@pwr.edu.pl (M.S.); pawel.zielonka@pwr.edu.pl (P.Z.); pawel.stabla@pwr.edu.pl (P.S.); grzegorz.lesiuk@pwr.edu.pl (G.L.)

**Keywords:** Multiaxial fatigue, CFRP, thin-walled structures, acoustic emission, initiation phase

## Abstract

The fatigue behavior of continuous fiber-reinforced composite materials is still not fully understood, particularly under multiaxial out-of-phase loading conditions. This study assesses the multiaxial fatigue behavior of thin-walled carbon fiber-reinforced polymer (CFRP) tubular specimens fabricated by filament winding (FW). A comprehensive experimental study is presented, investigating axial-torsion loads, phase shifts (0°, 45°, and 90°), and load ratios (−1, 0.05, and 0.5). Simultaneously, the acoustic emission (AE) method provides supplementary data for assessing fatigue damage accumulation. Consequently, a shear nonlinear material model and progressive damage in a shell-based finite element model were applied for stress analysis. The experimental results demonstrate the negative influence of a 90° out-of-phase load and the detrimental effect of mean stress for investigated positive load ratios. These findings offer valuable insights into the impact of phase shift (*δ*) and load ratio (*R*) in filament-wound carbon composites. These are essential for accurately modeling the fatigue behavior of composite materials under complex multiaxial loading.

## 1. Introduction

The European Union’s regulations aiming for a net-zero emissions balance by 2050 compel numerous industrial sectors to prioritize reducing CO_2_ emissions. This ambitious goal necessitates significant changes in energy consumption, production processes, and the adoption of sustainable technologies. It can be achieved by applying various solutions. Many industries, such as the civil engineering, automotive, and aerospace industries, extend the use of lightweight composite elements or structures to reduce costs, energy, and greenhouse gas emissions [1]. Their superior mechanical properties allow the design of increasingly complicated parts for more responsible applications. In addition, they are applied as a reinforcement structure to extend the durability of aging infrastructure throughout the world. The Boeing 787 is a shining example of a structure of 50% composite materials by weight (80% by volume). Additionally, it is the first airplane equipped with an airframe made of a composite material [2].

Developing composite materials in structural engineering requires a comprehensive and detailed design process that provides safety and reliability in service. This process is highly complex due to the multiaxial stress states caused by the anisotropy of the material (internal multiaxiality). This stress state is also compounded by multidirectional loads, i.e., external multiaxiality caused by at least two external loads acting at different planes. Frequently, the maximum and minimum stresses caused by loads do not coincide; this effect is caused by out-of-phase loading conditions. The impact of this phenomenon on fatigue life is well described for various metallic materials; however, experimental results are still required in continuous fiber-reinforced polymers. Understanding the fatigue behavior of continuous fiber-reinforced polymers subjected to external multiaxial loading conditions is essential in many applications (i.e., wind turbine blades, composite driving shafts, and high-pressure vessels) in order to accurately predict fatigue life. It can be assessed by reliable experimental characterization of material with a focus on factors affecting fatigue life, such as out-of-phase load and mean stress effect.

Multiaxial loading conditions, experimentally applied as combined tension–torsion, result in the complex stress states as experienced by components such as wings and turbine blades, which are subjected to aerodynamic loads that induce bending (i.e., tension and compression) coupled with torsion (shear) [3]. Olsson [4] presented a paper that reviews multiaxial experimental methods, suggesting that a tubular specimen is an optimal choice for investigating the tension–torsion cycling loading conditions and material systems used in cylindrical elements, such as elements manufactured by the filament winding method. Beneficially, this type of specimen avoids the free-edge effect. There are two standard approaches to manufacturing this type of sample: filament winding or mandrel wrapping, followed by curing in an autoclave [4]. Nonetheless, the multiaxial behavior of CFRP can also be investigated using a cruciform specimen, as demonstrated in Refs. [5,6]. The fatigue tests on cruciform specimens allow for applying only axial loads (tension–compression) in transverse and horizontal directions. However, the design of the cruciform specimen is challenging due to the requirement for damage initiation to occur in the gauge section. This necessitates reducing the thickness in the gauge section and adding a corner fillet to prevent premature failure outside the gauge section. In Ref. [5], the open-hole cruciform is designed to force damage initiation in the gauge section. This research revealed a sigmoidal stiffness decrease in cross-ply and quasi-isotropic laminates. Moreover, machines designed for biaxial cruciform specimens are highly complex, requiring at least two actuators and a suitable control system with a feedback loop.

Mei [7] provided an example of systems where out-of-phase loading conditions occur, such as the loading spectrum recorded for the lower wing skin of an aircraft, which exhibits a phase shift between stress components. Considering the Boeing 787, whose wings are primarily made from carbon fiber-reinforced polymer (CFRP), there is a genuine need to examine the impact of phase shifts on CFRP material. It is also worth noting that CFRP is frequently used in the aerospace, military, and wind power industries [8]. The scientific literature survey on composite materials shows a shortage of research on the multiaxial fatigue performance of those materials, specifically under out-of-phase load [9,10,11]. The number of research groups focused on the multiaxial fatigue behavior of composite materials remains limited, primarily due to the high costs and time-intensive nature of such experiments, which require rigorous testing protocols and specialized equipment.

The fatigue behavior of glass fiber-reinforced polymer (GFRP) has been extensively studied in Refs. [3,9,12,13,14,15,16,17], particularly regarding factors influencing fatigue life. Ref. [14] discusses the detrimental effects of the shear stress component and biaxiality ratio on transverse fatigue strength and crack initiation. Additionally, Ref. [15] presents results on the propagation phase, where the crack growth rate was found to increase significantly with the biaxiality ratio (λ_12_ ≥ 1). The impact of the load and biaxiality ratio on crack initiation and propagation under tension-torsion loadings has been revealed in Ref. [17], which shows that the shortest fatigue life occurs for *R* = −1. A study on internal multiaxiality resulting from material anisotropy is presented in Ref. [16], where a comparison is made between fatigue damage in multidirectional laminates (internal multiaxiality) and tubes subjected to external multiaxial loading conditions. The results indicate that damage evolution is comparable when the biaxiality ratios are similar. This research suggests that multiaxial fatigue behavior can be effectively investigated using flat coupons by taking advantage of internal multiaxiality—i.e., the local multiaxial stress state induced by material anisotropy. When considering flat coupons, all three stress components are present simultaneously, which makes it impossible to decouple their individual effects [3]. In this context, the tubular specimen remains the most effective geometry for investigating the multiaxial response of composite materials. It should be noted that the results presented in this paragraph primarily focus on GFRP materials under in-phase loading. However, there remains a niche for exploring other composite materials, produced using different manufacturing technologies, under out-of-phase loading conditions.

The experimental data for CFRP material under in-phase and out-of-phase loads are given in Ref. [10]. It shows the application of the critical plane energy-based parameter in multiaxial fatigue life prediction. The presented experimental data show the limited impact of phase shifting for the following stacking sequence of laminate [45/70/0/−70/−45]. The critical plane approach is also investigated in Ref. [18]; however, the effective stress parameter is applied to describe the damage state in the investigated GFRP material system. The results show that the winding angle (i.e., fiber orientation of the lamina) has a significant influence on the fatigue life of filament-wound tubes, with shorter fatigue life observed at higher fiber angles.

Experimental data from the literature are summarized in Table 1, which lists the collected papers along with key information, including matrix, fiber type, load ratios, lay-up configuration, and specimen type.

Some factors, such as the lack of material’s transparency, hamper research on CFRP material. The damage observation is limited since the fibers are not transparent, and a more sophisticated method is needed to assess the damage. The transparency of the GFRP material allows for a precise observation of the damage that appears in the material. Among the techniques utilized, acoustic emission (AE) has been extensively used due to its advantages in in-situ damage monitoring with high sensitivity and its capability to continuously inspect a relatively large area [31]. However, due to the complex failure nature of composite materials, defining primary failure modes is extremely challenging because multiple failure mechanisms can act simultaneously. A comprehensive review in Ref. [31] shows the damage characterization using acoustic emissions. Several parameters are used in the literature for damage initiation detection, i.e., cumulative curves of AE futures or a combination of AE and mechanical data. Some possibilities of using more advanced techniques, i.e., machine learning for assessing the damage, are presented in Refs. [32,33,34,35].

**Figure 1:**
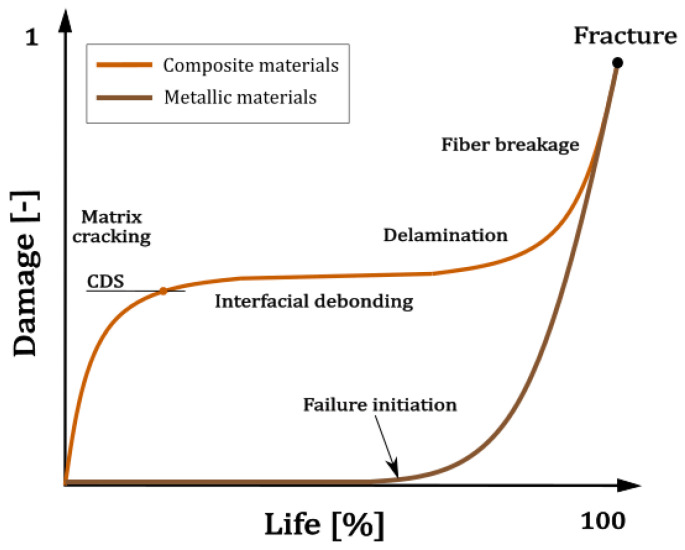
The general nature of damage accumulation in composite materials and metallic materials. Adapted from [36], Elsevier, 2002.

The evolution of damage in composite materials differs from the accumulation process in metallic materials, as illustrated in Figure 1. (redrawn based on [36]). Regarding composite materials, the curve passes through three stages associated implicitly with a particular failure mechanism. The initial stage is dominated by the cracking of the matrix, and its density increases until the point of the characteristic damage state (CDS) is reached. This point on the curve represents the beginning of interfacial debonding (due to the high crack content). During the same time, local delamination and fiber breakage may occur. The progression of interfacial debonding leads to delamination, which, in the final stage, in combination with large-scale fiber breakage, is expected to lose the integrity of the laminate [37]. Progressive fatigue failure is the main objective investigated in Refs. [38,39,40,41], both numerically and experimentally. The fatigue damage model (FDM) developed in these works is based on an energy-based approach and has shown good predictive capabilities under various loading conditions and material configurations.

The reviewed literature allows for a few general conclusions. Although some research has addressed out-of-phase multiaxial fatigue, a deeper understanding of the fatigue behavior of fiber-reinforced materials under multiaxial loading conditions is still required. In particular, more effort is needed in the analysis of carbon-fiber-reinforced polymers (CFRPs) manufactured using filament winding technology, which are widely used in applications such as pressure vessels. Most existing studies on out-of-phase or multiaxial fatigue have focused on ply- or specimen-scale tests of glass-fiber-reinforced composites produced from prepregs, while similar investigations for filament-wound CFRPs are scarce.

The presented research concerns the experimental-numerical characterization of the multiaxial fatigue behavior of thin-wall tubular CRFP structures under in-phase and out-of-phase axial-torsion loading conditions. This study presents a more advanced stage of the research, extending the static analysis introduced in Ref. [42], which focused on quasi-static material characterization and numerical modeling toward the investigation of fatigue behavior. Evaluating CFRP multiaxial behavior through acoustic events and numerical analyses shows the investigation’s innovative nature, providing a comprehensive database for fatigue life prediction approaches.

## 2. Experimental Program

The experimental section focuses on the mechanical conditions established for in-phase and out-of-phase axial-torsion fatigue investigation and measurement methods applied to characterize the behavior of the cylindrical thin-walled CFRP tube. An axial-torsion loading is applied using a biaxial servo-hydraulic testing machine, while a piezoelectric sensor simultaneously collects the signals (acoustic waves in the material) generated during cyclic loading. The experimental data evaluate the influence of the phase shift effect and mean stress on fatigue life in the high-cycle regime.

### 2.1. Material and Test Specimen

This investigation aims to determine the mechanical fatigue performance of the CFRP tube under cyclic multiaxial loading conditions, which have already been tested under quasi-static loading conditions in Ref. [42]. The static tensile tests exhibited a coefficient of variation below 10%, indicating relatively consistent material behavior under monotonic loading. The following constituents were used to manufacture CFRP thin-walled cylindrical structures: carbon fiber (CF) ZOLTEK^TM^ PX35 and Araldite LY1564 epoxy resin with Aradur 3474 hardener. SEM images were used to assess the fiber volume content, which was found to be 55%, based on computer image analysis. The micromechanical approach by Abolin’sh [43] was applied to estimate the effective elastic properties of the CFRP tube, as presented in Table 2. This data has already been published in Ref. [42] and is incorporated into the research.

**Figure 2:**
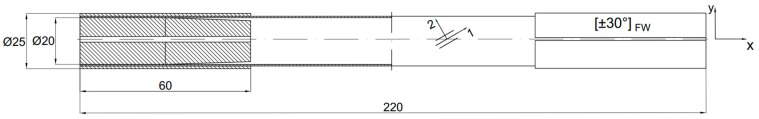
Dimensions of the test specimen used for experimental multiaxial fatigue characterization.

The tubular specimen is a reasonable choice regarding global multiaxial loading conditions. It excludes the undesirable effect of free edges, causing stress concentration, which results in delamination. For this research, the fiber orientation was chosen at 30°, and filament winding technology was applied to manufacture thin-walled CFRP tubes of two layers with a mosaic pattern of 1/1 [42]. In the process, 20 mm diameter chrome-coated steel mandrels were applied. Before each winding process, it was necessary to cover the mandrels with wax and polish them to facilitate the demolding process. Special shrink tape was used to cover the wet surface of the sample to remove resin overflow and provide sufficient smoothness of the external surface. After the winding process, the composite tubes were rotated and simultaneously cured at room temperature. After demolding, the tubes were cut into samples, and a post-curing process was applied according to the resin producer’s recommendations (1 h at 80 °C and 4 h at 120 °C). The specimen dimensions and geometry are presented in Figure 2. Laminate and material axes of reference are labeled as *xyz* and *123*, respectively.

The geometry and fiber orientation were analyzed, including the 3D scanning method. Referring to the assumed fiber orientation (±30°), a discrepancy with a standard deviation of 0.45° was observed. The nominal stresses were calculated as:

(1) $$\sigma_{x}=\frac{F}{{2\pi r}_{m}t},$$

(2) $$\tau_{xy}=\frac{T}{2\pi r_{m}^{2}t},$$

where *F* is the axial force, *T* is the torsional load, *t* is the thickness, and *r_m_* is the mean radius of the specimen. To calculate geometrical stress, the outer diameter needed to be measured. It was estimated by measuring the sample mass and length and applying the material density (0.00152 g/mm^3^), providing a mean value of 21.894 mm with a standard deviation of 0.091 mm. Calculated nominal stress does not represent the actual stress state in composite materials, as they are calculated on a macroscale over a homogenized area, which excludes layup configuration, i.e., fiber orientation. Nonetheless, they were used to control the fatigue experiment and demonstrate the impact of the load radio and phase shift.

### 2.2. Multiaxial Fatigue Tests

**Figure 3:**
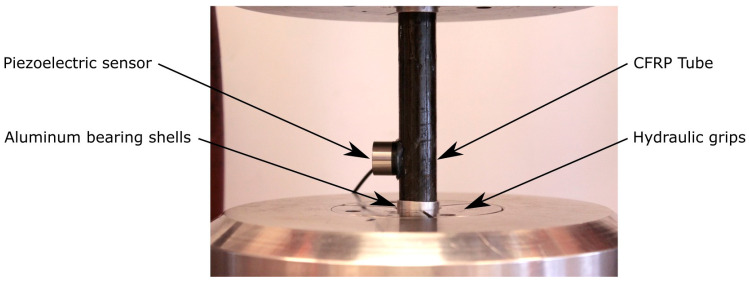
Experimental setup used for providing multiaxial loading conditions and acoustic signal acquisition.

Experimental investigation focuses on thin-walled tubes subjected to axial-torsion loads under reference proportional (in-phase) and non-proportional (out-of-phase) loads. Various load ratios (*R*) with a constant biaxiality (*λ_T_*) and a frequency of 6 Hz were applied to the specimens using the MTS 809 axial-torsion test systems equipped with a calibrated class 1 load cell. The in-situ surface temperature was controlled using the thermal camera at constant intervals. Additionally, an acoustic emission system was applied to acquire acoustic waves caused by failures within the material. This method was used to analyze the CFRP cumulative curve of failure events. The experimental setup is illustrated in Figure 3.

The multiaxial fatigue behavior was investigated under the following test parameters:

- Phase shift (*δ* = 0°, 45°, 90°) considering the applied sinusoidal signals for axial-torsion loads.
- Constant biaxiality ratio (*λ_T_* = 1), calculated based on Equation (3).

(3) $$\lambda_{T}=\frac{\tau_{xy,\;amp}}{\sigma_{x,amp}},$$

where $\tau_{xy,a}$ and $\sigma_{x,\;a}$ are the amplitudes of the nominal shear and normal stresses, respectively [MPa].

- Load ratio (*R* = −1; 0.05; 0.5) is defined as *R = F_min_/F_max_ = T_min_/T_max_*, where *F*—axial force and *T*—torque.

**Figure 4:**
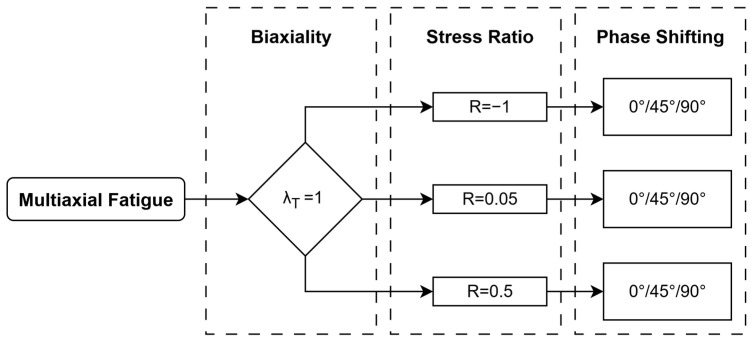
Flow chart of the experimental parameters investigated.

The flow chart of the experimental parameters investigated is illustrated in Figure 4. The load-control fatigue tests were performed until decohesion of the specimen occurred (failure through thickness) or were aborted at the run-out value of 2 × 10^6^ cycles. The presented experimental campaign included a total of 110 specimens tested under three different *R*-ratios: −1, 0.05, and 0.5, corresponding to 38, 36, and 36 specimens, respectively. Among them, 37 were tested in-phase, 39 with a 45° out-of-phase shift, and 34 with a 90° out-of-phase shift.

The Vallen AMSYS-6 system collected acoustic signals by a piezoelectric VS150-M sensor with a frequency range of 50 to 600 kHz. Due to the surface curvature, the sensor was mounted to the sample using hot glue, which acts as a waveguide and adhesive, as shown in Figure 3. It allows for properly transferring the elastic waves from the material to the sensor, verified by a pencil lead break test.

## 3. Numerical Analysis

Section 3 includes a description of the discrete model and a stress analysis of the FW tube using the finite element method. This investigation incorporated the numerical model, which accounts for the material shear nonlinearity and progressive damage, as previously presented in Ref. [42]. Compared to the referenced study, the numerical analysis in this section is extended by incorporating buckling analysis.

### 3.1. Numerical Model

**Figure 5:**
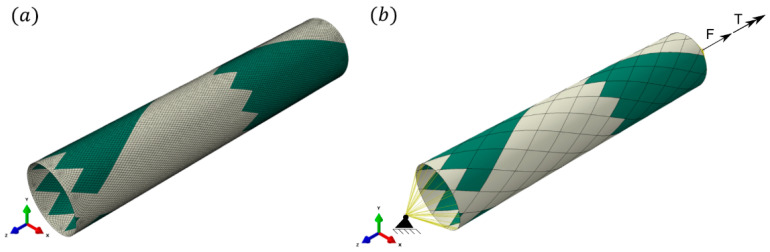
(**a**) Discrete model of the investigated specimen; (**b**) section assignment with diamond created in the winding process (different colors reflect various fiber angles); and a characteristic area on the border between various fiber angle layers called zig-zag is visible. Adapted from [42], Elsevier, 2024.

The applied discrete model, material model, and progressive damage are described in detail in Ref. [42]. Therefore, only crucial aspects of the numerical model are highlighted. The tubular specimens investigated in this research were manufactured in the FW process, resulting in the complex interwoven structure of the fibers, which provides various mosaic patterns. In Refs. [44,45,46,47,48], a significant effect of the mosaic pattern on the mechanical performance of the FW tubes is shown. It justifies including the pattern in the geometrical model, especially if the number of composite layers is limited. The geometrical shell model was created using the script developed by the authors (a detailed description can be found in Ref. [44]). Figure 5 presents the geometry with an applied mesh of 18 855 S4R elements and the assignment of sections using the applied geometric modeling approach of the authors (green and beige colors indicate different fiber orientations). Considering material architecture, the triangular-shaped border between different colors is called zig-zag, which is a significant area for stress concentration due to the stiffness gradient caused by a sudden change in the orientation of the fibers. An underestimation of stresses occurs if the numerical model does not account for areas of fiber undulation (zig-zag) [49,50,51].

The mechanical behavior of the specimen is described by the constitutive equation in Equation (4) provided by Chang [52]. It reflects the nonlinear stress–strain relationship due to the static accumulation of damage in shear, which is expressed by the damage parameter *d* given by Equation (5). The applied approach combines nonlinear shear material behavior with progressive damage, incorporating stress and failure analysis of the laminate. Stress and strain were computed using nonlinear finite element analysis based on finite deformation theory, accounting for both material and geometric nonlinearities [52]. Damage was introduced by reducing specific material properties. In Abaqus, this was implemented using the * USER DEFINED FIELD option and the USDFLD user subroutine.

(4) $$\tau_{12}^{\left(i+1\right)}=\left(1-d\right)G_{12}\gamma_{12}^{\left(i+1\right)}$$

(5) $$d=\frac{3\alpha G_{12}{\left(\tau_{12i}^{\left(i\right)}\right)}^{2}-2\alpha {\left(\tau_{12i}^{\left(i\right)}\right)}^{3}/\gamma_{12}^{\left(i\right)}}{1+3\alpha G_{12}{\left(\tau_{12i}^{\left(i\right)}\right)}^{2}}$$

where *i* represents the increment number, *G_12_* is the (initial) ply shear modulus, *d* is the damage parameter characterized by Equation (5), and *α* reflects the material shear nonlinearity, which refers to the fourth-order constant in polynomial function of strain energy density [53,54] set for 2.44 × 10^−6^ [MPa^−3^] based on Ref. [42]. Three damage states described by the equations given in Ref. [42] govern the progressive failure of the FE model, i.e., fiber-matrix debonding, matrix tensile cracking, and matrix compressive failure. The numerical model also considers fiber buckling to be a catastrophic failure mode. After geometrical instability occurs, the material can no longer support any loads. It occurs when the maximum compressive stress in the fiber direction exceeds the fiber buckling strength (*X_c_*). The applied strength parameters to describe the highlighted failures are presented in Table 3 and have been taken from Ref. [55].

The applied boundary conditions are presented in Figure 5b. At reference point no. 1 (RP1), all translations and rotations are constrained (set to zero). A combined load consisting of a force ($\bar{F}$) and a torque ($\bar{T}$) was applied on the opposite side of the specimen by prescribing a displacement and a rotation angle along the *z*-direction. In the model, auxiliary coordinate systems were created to enhance stress analysis along the principal axis (material coordinate system) for given composite layers. By utilizing this approach, the proper stress analysis was conducted.

### 3.2. Stress Analysis

**Figure 6:**
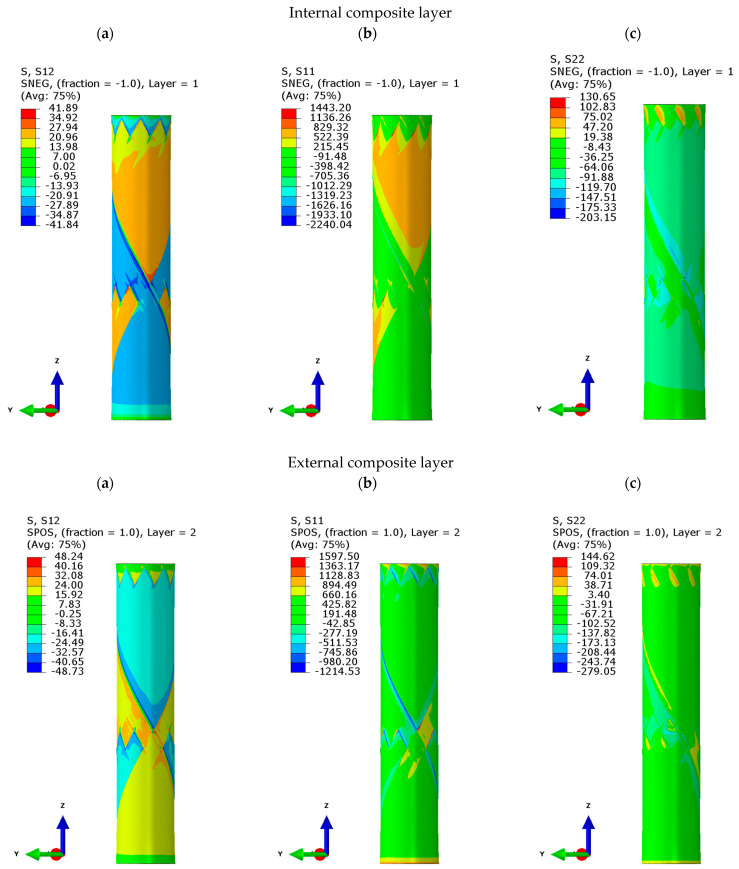
The finite element analysis of a tubular specimen under the multiaxial load (*F* = 13.9 kN, *T* = 74.1 Nm) for the internal and external lamina, showing: shear stress (**a**) $\tau_{12}$, and normal stresses (**b**) $\sigma_{1}$ and (**c**) $\sigma_{2}$ in MPa.

Finite element analysis was performed to evaluate stress distribution under combined loading, i.e., static axial-torsional. The stress tensor components for both the internal and external composite layers are given in Figure 6. This analysis provided a preliminary overview of the mesoscale stress distribution and, consequently, the likely area of final fracture.

**Figure 7:**
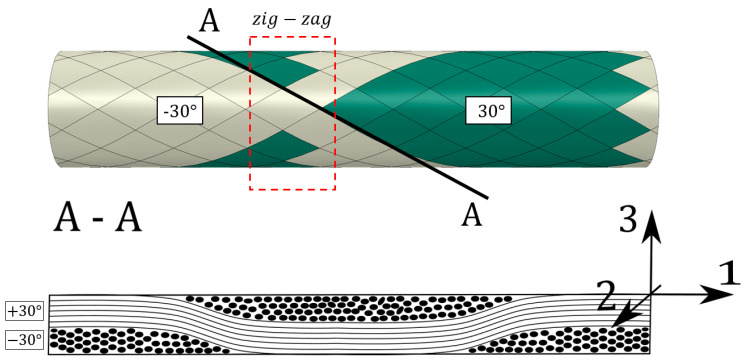
Schematic representation of the zig-zag area.

The FEA reveals an inhomogeneous stress distribution resulting from the mosaic pattern, where each lamina consists of two opposing fiber orientations. Significant stress concentrations occur in the zig-zag region due to fiber undulation and abrupt changes in fiber orientation, which create a steep stiffness gradient. A cross-section of this area is shown in Figure 7. The local stress concentration along the fiber bundle border can also be observed, especially for shear stress (*τ_12_*). The presented analysis reveals that those areas (zig-zag) will play a critical role in the initiation and progression of fatigue damage.

### 3.3. Buckling Analysis

**Figure 8:**
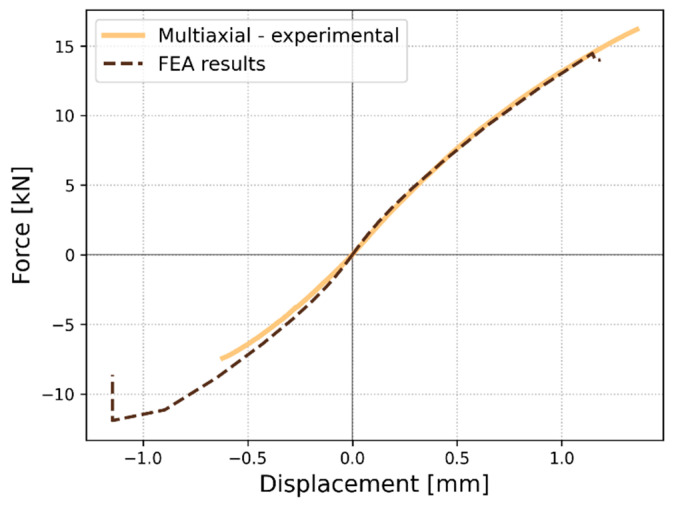
The average force-displacement curve for the quasi-static axial-torsional loading case is presented along with the numerical results.

A fatigue experiment under *R* = −1 is considered within the research framework. In the case of composite materials, a compression load often raises many doubts; one of the critical issues is the buckling phenomenon acting at various length scales. This phenomenon influences fatigue behavior by significantly reducing fatigue lifetime. The reasons highlighted raised the need to conduct the compression-torsion experimental test to investigate the global stability of the structure. At the same time, a numerical analysis of nonlinear buckling was run to reflect the global buckling behavior of the material using the static Riks analysis [56]. The results corresponding to the quasi-static testing and FEA are presented in Figure 8. The experiment under compression load gives a maximum value of 7.43 kN ± 0.33 kN, whereas the developed numerical model presents a maximum value of 11.91 kN. This discrepancy might be easily explained since the created discrete model does not include microstructure imperfections. Since composite materials are susceptible to compressive load, the compressive strength is highly dependent on the discontinuity of the microstructure. However, a good stiffness agreement is observed for the applied material model.

The conducted Riks analysis was devoted to predicting the geometrically nonlinear collapse of a structure, delivering the critical force value of 11.91 kN, indicating that global stability is maintained up to compressive strength. The experimental maximum axial force is 7.43 kN. This FE analysis allows the assumption that the critical force for global buckling is equal to the experimental value. The maximum compressive load applied during fatigue totals 2.6 N, which gives a safety margin of approximately 2.86.

**Figure 9:**
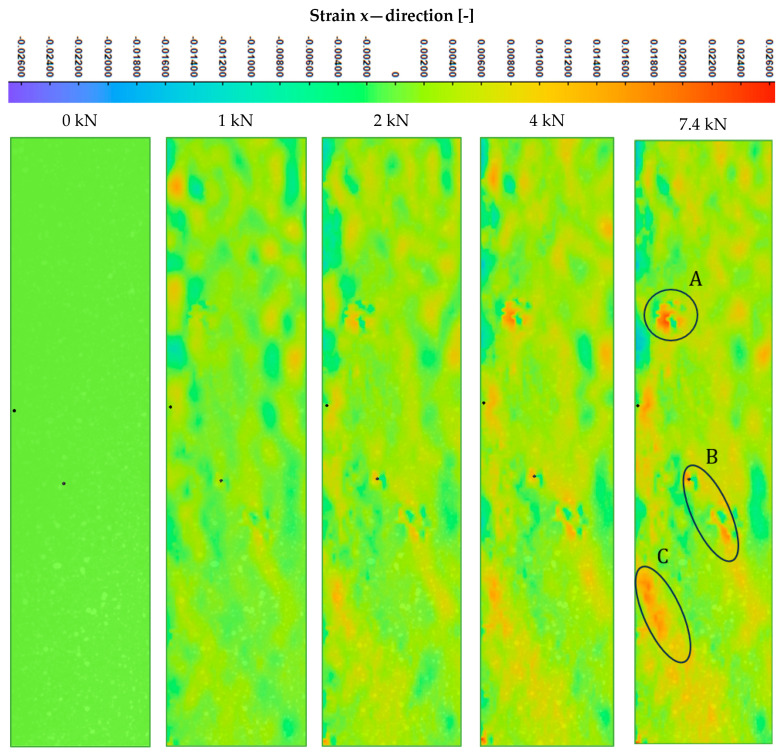
Strain fields evolution during the quasi-static compression-torsional test measured using the DIC system.

The experiment was carried out with the digital image correlation system (DIC). It provided the strain fields at particular load intervals; the obtained results are presented in Figure 9. The local strain concentration can be observed in areas A, B, and C. The buckling phenomena can be observed at various length scales, i.e., macroscale or mesoscale (global buckling) or microscale (kinking or local buckling). The numerical analysis provided a result at the mesoscale for global buckling: there is no instability in geometry before reaching the maximum compressive force value; however, the instability at the microscale cannot be excluded from consideration based on the numerical and DIC data.

## 4. Results and Discussion

This section discusses the multiaxial fatigue behavior of CFRP material. It starts with analyzing the S-N curves for various phase shifts (0°, 45°, and 90°) and load ratios (−1, 0.05, 0.5). Subsequently, the stiffness curves based on displacement and rotation amplitudes are analyzed. Using the study of acoustic signals, S-N curves corresponding to the damage state at the CDS are derived and compared with the total fatigue life.

### 4.1. Multiaxial Experimental Data

The presented S-N curves are fitted using linear regression in the double logarithmic scales according to ASTM E739 [57], which uses the power law (Equation (6)).

(6) $$N_{f}=A\sigma_{x,amp}^{B}$$

where *N_f_*—life, *σ_x_*_, *amp*_—geometrical stress amplitude, and *A*, *B*—function parameters. Regression analysis was performed on fatigue data, considering stress as an independent variable. All experimental data are presented with the scatter bands of 5.

In-phase load

**Figure 10:**
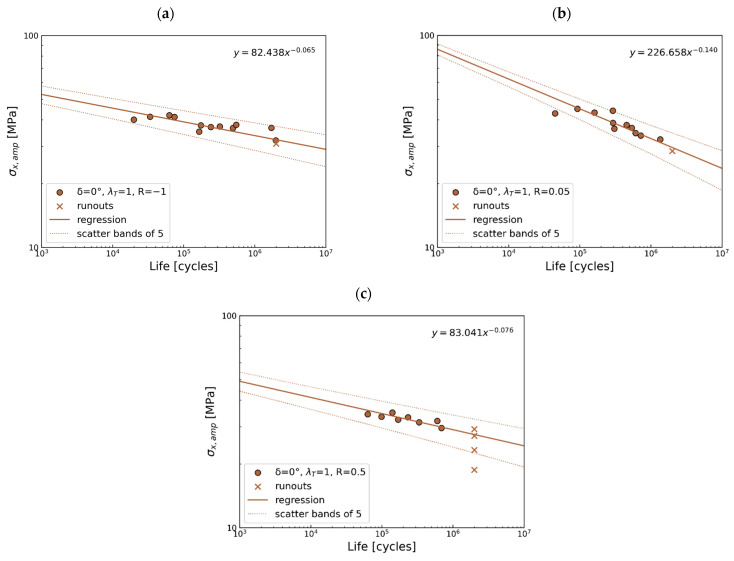
Multiaxial fatigue S-N data for proportional loading for (**a**) *R* = −1, (**b**) *R* = 0.05, and (**c**) *R* = 0.5.

Considering the S-N plots in Figure 10, for *R* = −1 and 0.5, the band lines cover all points. On the other hand, a larger scatter of results can be observed for *R* = 0.05. In this case, 1 of 11 points is out of the scatter band 5. The slope for the regression lines is approximately similar to *R* = −1 and 0.5, whereas *R* = 0.05 is much larger.

45° out-of-phase

**Figure 11:**
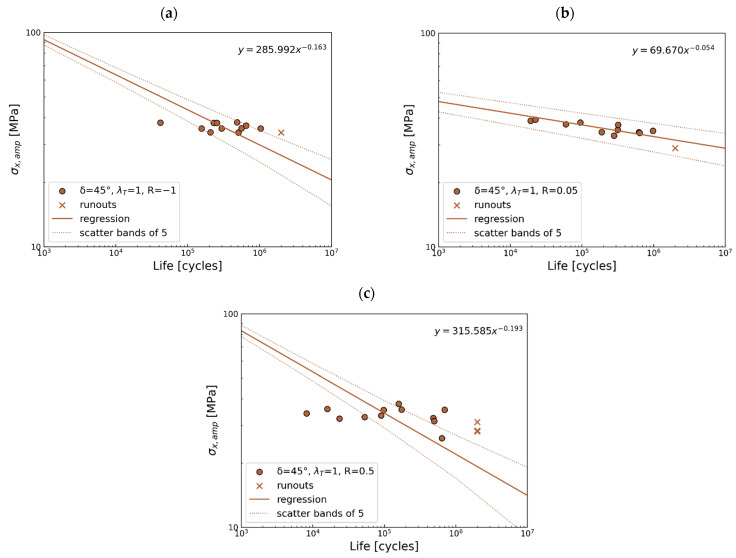
Multiaxial fatigue S-N data for phase shift 45° for (**a**) *R* = −1, (**b**) *R* = 0.05, and (**c**) *R* = 0.5.

Regarding S-N curves in Figure 11, for *R* = 0.05, all points are within the scatter band. For *R* = −1, only one is outside the scatter band, and the largest scatter of the results exhibits *R* = 0.5, where 7 points out of 12 are not covered by the scatter band of 5. In the case of this data, the slopes are divergent. Considering Figure 11a,c, the experimental data exhibits a horizontal trend, while the statistical analysis gives a highly decreasing trend, giving a large scatter. These experimental data indicate that, under the present loading conditions, stress level alone does not determine fatigue life, as a single stress level produces a wide range of fatigue lives. Such effects may arise from manufacturing-induced defects and their orientation relative to the applied loading, which can be particularly sensitive to out-of-phase conditions. Therefore, the initial state of the material may play a crucial role under 45° out-of-phase loading.

90° out-of-phase

**Figure 12:**
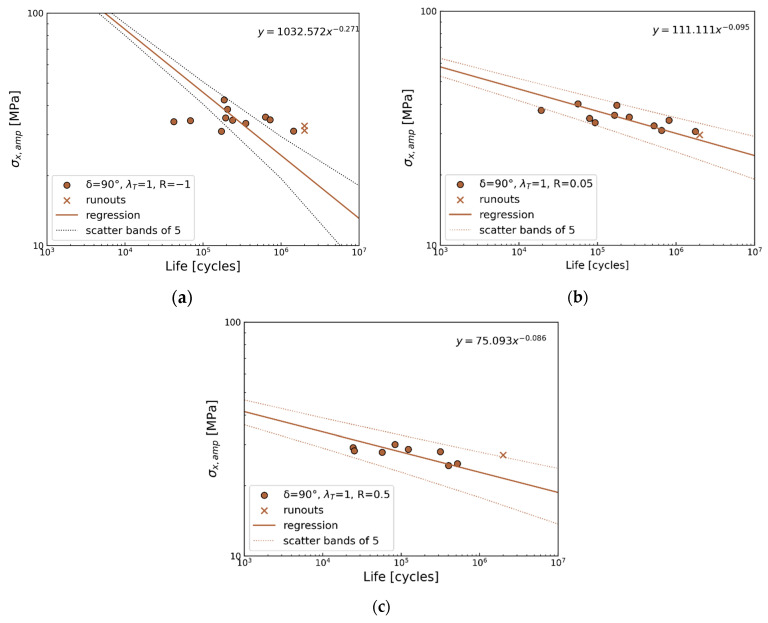
Multiaxial fatigue S-N data for phase shift 90° for (**a**) *R* = −1, (**b**) *R* = 0.05, and (**c**) *R* = 0.5.

The reverse load ratio *R* = −1 exhibits the largest scatter in this group; six points are out of the scatter band, and the remaining data for positive load ratios exhibit scatter within the band of 5. Similar to the previous observation (Figure 12a,c), a horizontal trend is seen in Figure 12a, while the statistical analysis shows an opposite trend. In these cases, the focus should be placed on the experimental data rather than the regression line. It should be noted that the *R*-ratio of −1 is particularly challenging due to the presence of compressive forces, which result in fatigue behavior differing significantly from that under tensile loading [53].

The regression coefficients of determination (*R^2^*) for each experimental dataset are presented in Table 4. Analyzing the *R^2^* parameter, low correlations (0.03, 0.20, 0.26) between the regression and the experimental data are observed for the 45° out-of-phase load, suggesting high scatter and limited predictive capability of the regression in these cases. A moderate correlation (0.56, 0.40) is observed for *R* = −1, suggesting only partial correlation and a moderate level of regression reliability. Considering the trends and scatter of the experimental data, the role of manufacturing must be emphasized. The manufacturing process plays a crucial role in composite materials, as it defines the resulting microstructure, which often includes defects such as irregular fiber distribution, fiber misalignment, matrix voids, and unbonded interfaces. These imperfections introduce variability in the initial material state, leading to different conditions for damage initiation and progression toward critical failure, ultimately contributing to the scatter observed in fatigue life [54].

**Figure 13:**
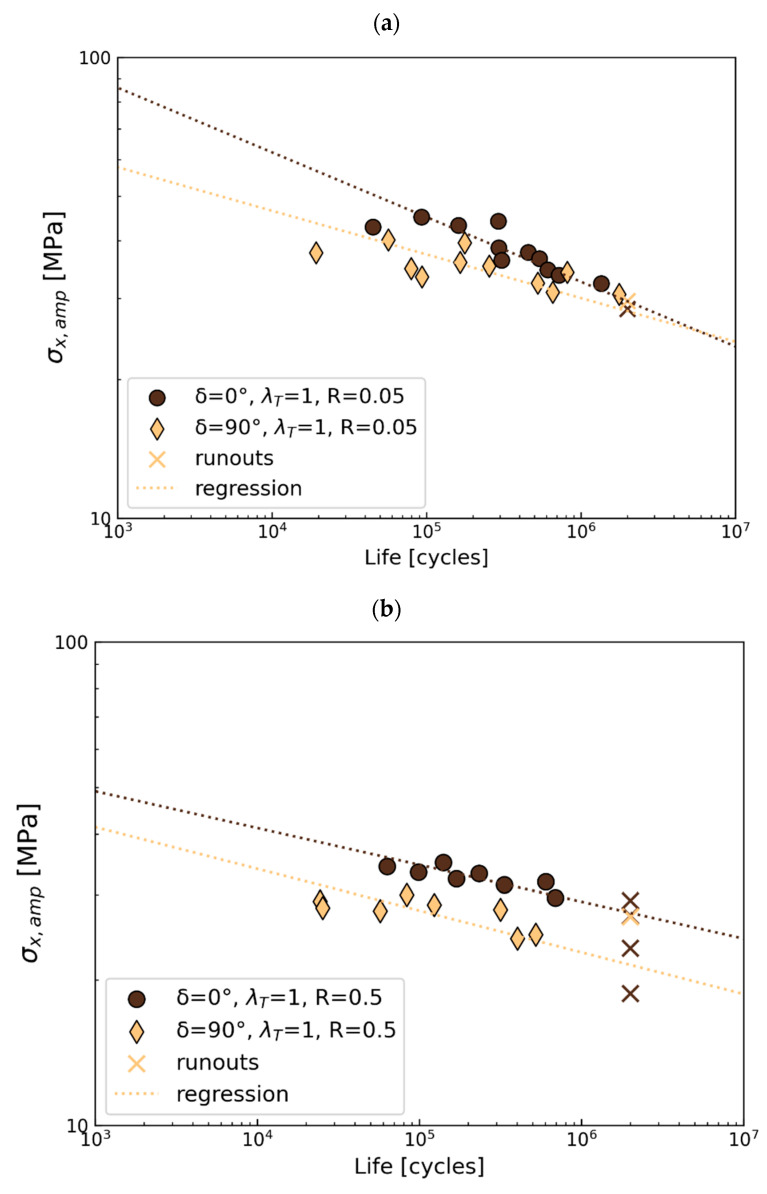

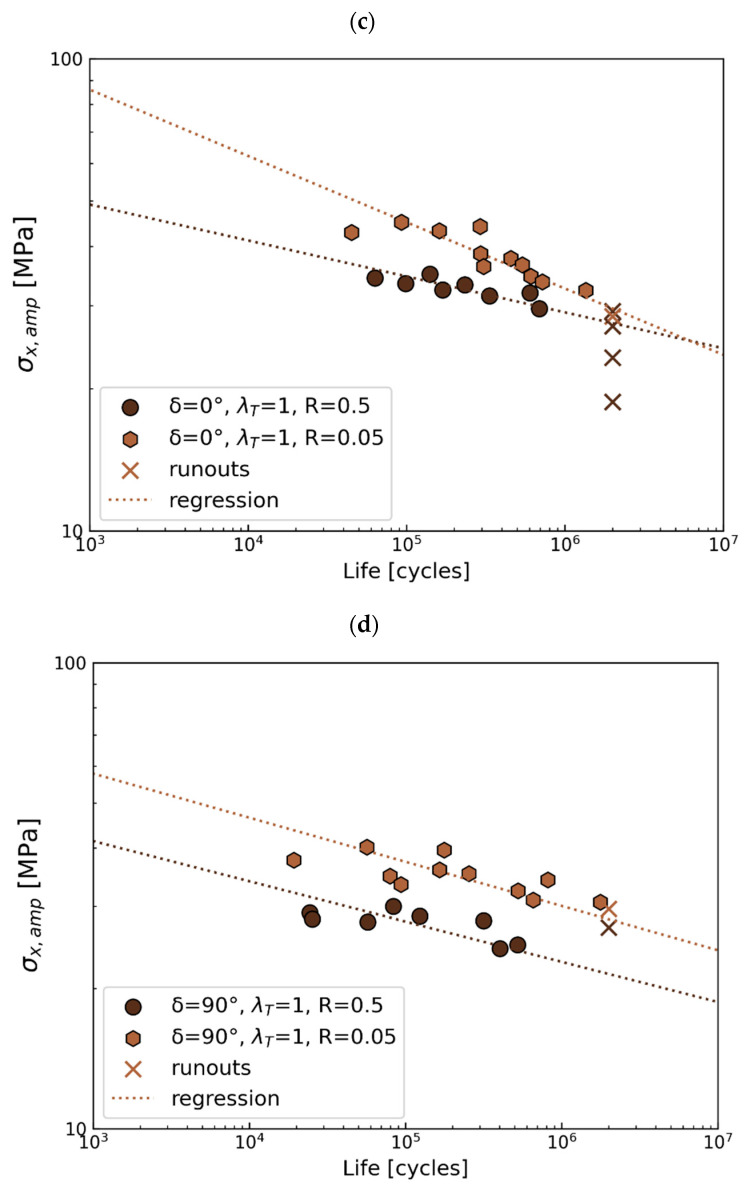
Phase shift effect on S-N data for (**a**) *R* = 0.05 and (**b**) *R* = 0.5. Impact of mean stress on fatigue data; (**c**) proportional load, (**d**) out-of-phase load.

Due to the horizontal trends observed and the low correlation for the negative load ratio and the 45° out-of-phase loading, the influence of mean stress and phase shift cannot be clearly identified. Consequently, the influence of the investigated parameters (phase shift and mean stress) is presented and discussed only for in-phase and 90° out-of-phase loading conditions with positive load ratios, as shown in Figure 13.

In Figure 13a, the regression slopes differ, and the reference (in-phase load) regression exhibits a higher slope than for the out-of-phase load; however, a negative impact of phase shift in this load ratio can be recognized, and it is significantly pronounced within a short fatigue life. A decrease in fatigue strength under out-of-phase load is also observed in Figure 13b for a load ratio of 0.5. From the phenomenological point of view, a negative effect of 90° out-of-phase load on fatigue strength in the high-cycle fatigue regime is observed.

In addition to the impact of the phase load, the influence of the mean stress is considered for in-phase and out-of-phase loads. The effect of mean stress is noticeable; a higher load ratio (*R* = 0.5) gives a shorter fatigue life in both cases. These results contrast with the literature, where a higher mean stress (*R* = 0.5) is associated with a longer fatigue life compared to a lower mean stress (*R* = 0.05), as shown in Ref. [17]. However, it is important to clearly state that the manufacturing technology of the specimens differs. In Ref. [17], mandrel wrapping and autoclave curing were used, while in the current study, the filament winding method was applied, resulting in a more complex microstructure with fiber undulations. Considering the effect of mean stress, the role of fiber undulations (zig-zag patterns) should be emphasized. Due to the filament winding process, these regions exhibit pronounced stress concentrations resulting from the large stiffness gradients between interlaced layers. In these regions, the composite layers compress one another, inducing localized compressive stresses that are directly correlated with the applied mean stress. Elevated mean stress levels in these areas promote increased damage due to compression and may significantly contribute to the observed reduction in fatigue performance. The experimental data provided emphasize the importance of a damage-based explanation and consideration of the phase shift and the mean stress effect in predicting the fatigue life of FW CFRP tubular members.

### 4.2. Stiffness Variation

**Figure 14:**
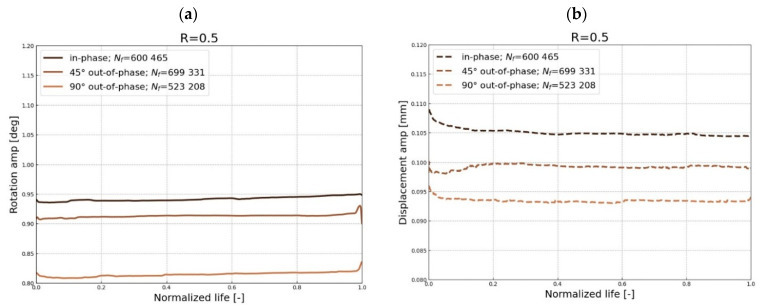

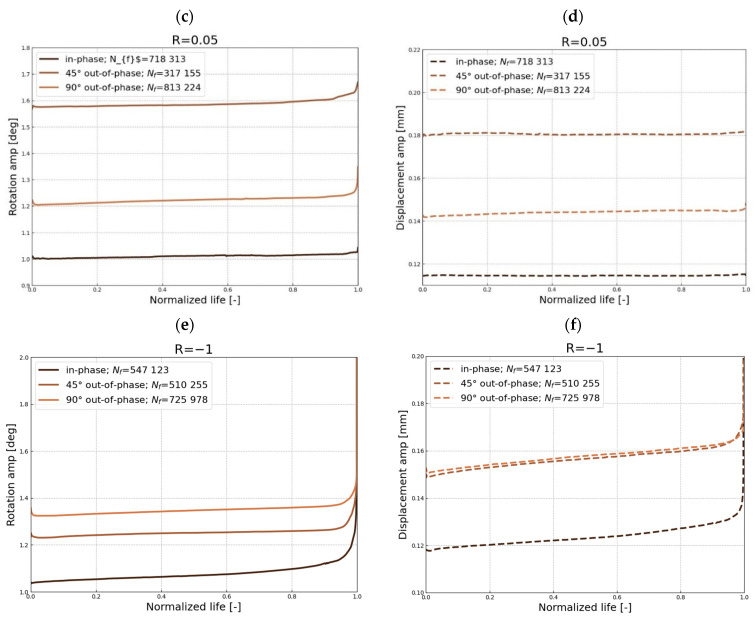
Displacement (dotted) and rotational (solid) curves as a function of normalized cycles acquired during the experimental campaign for *R* = 0.5 (**a**,**b**), *R* = 0.05 (**c**,**d**), and *R* = −1 (**e**,**f**).

The stiffness variation plotted in Figure 14 is analyzed based on the displacement and rotational amplitude acquired during the fatigue tests. Several factors can be associated with the stiffness variation in engineering materials, i.e., temperature effect, cycling softening or hardening, damage development, or the load exceeding the material limits. The cyclic softening/hardening is a material behavior unrelated to the damage; based on the obtained experimental data and literature study, there is no basis for considering this, and the remaining facts besides the damage development.

The stiffness degradation process is shown in Figure 14; in general, it can be divided into three stages. Initially, the stiffness increases progressively within several cycles; this stage is associated with the tailoring of the material and gripping specific loading conditions, which might also be governed by material state, i.e., defect content. This phase is clearly visible in Figure 14b, and lasts up to 15% of total life, while in the remaining cases, it is much shorter, lasting up to a maximum of 5%. The next stage encompasses the majority of the fatigue life and is mainly associated with microdamage such as debonding and matrix cracking. In this part, global stiffness gradually decreases as a result of the development of these failure mechanisms, as in Figure 14a,c,e,f [54]. In the remaining cases, the stiffness is either constant or increasing, as shown in Figure 14b,d. However, even though the axial stiffness remains constant or locally increases, the torsional stiffness still exhibits a gradual decrease. The literature acknowledges that the stiffness degradation process depends on both the evolution of matrix crack density driven by off-axis crack initiation and the length of nucleated cracks across the laminate width [15]. In the presented cases, the horizontal nature of the displacement and rotational curves indicates that the majority of fatigue life is governed by damage development on the microscale.

The macroscale damage propagation can be observed by the progressive drop of the stiffness in the last hundred cycles to the final failure. Thus, the progressive part of the curves shows the unstable propagation phase for this kind of specimen governed by delamination and fiber breakage. The layup configuration of the specimen supports the statement that the macro damage propagation proceeds rapidly within several cycles due to the lack of constraint layers (i.e., cross-ply layers), which could transmit the load while damage is developing at the off-axis composite plies. Therefore, the delivered fatigue curves are related to the initiation of the damage.

### 4.3. Analysis of the Acoustic Signals

**Figure 15:**
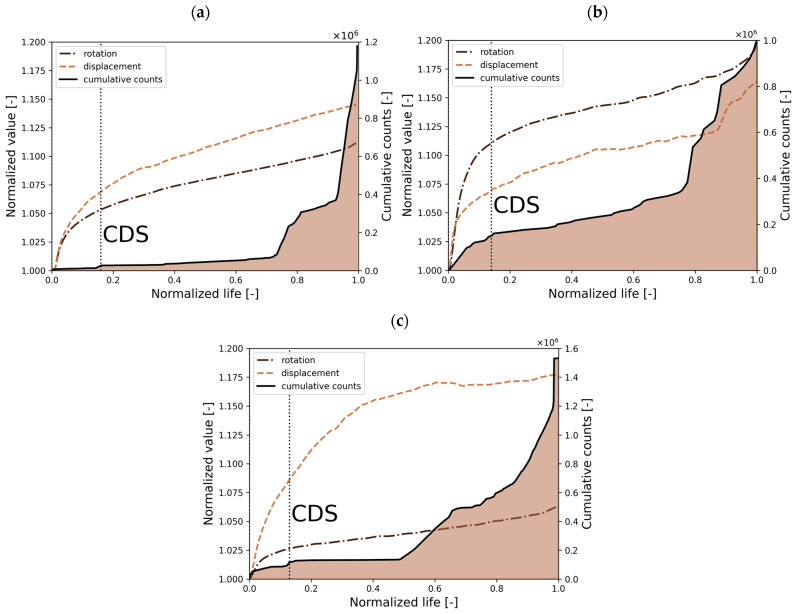
Normalized displacement and rotation correlated with cumulative counts (AE signals) as a function of normalized fatigue life for (**a**) in-phase, (**b**) 45° out-of-phase, and (**c**) 90° out-of-phase load under load ratio 0.5.

The acoustic emission (AE) system acquires the elastic waves that appear in the material by the piezoelectric sensor. Each failure within the material causes an elastic wave, which can be collected and described by the vector of parameters, i.e., frequency, energy, rise time, amplitude, etc. In the research presented, an analysis of cumulative counts recorded by the sensor was chosen and performed. Among the various parameters of the acoustic waves, this one was selected as an initial, general indicator of damage in the material. Although constant background noise may be present, an observed increase in the count gradient signals the onset of material failure, which in some cases can render the material unfit for further service. Based on these data, the relationship between cumulative counts and normalized life was established. One of the limitations of this approach is that the total number of recorded signals includes not only material-related damage events, but also background noise, such as friction in the grips or vibrations and sounds from the hydraulic testing machine. However, the group of signals analyzed was limited by filters of frequency and amplitude based on the values in Ref. [28]. The ultimate objective of this analysis is to identify the CDS point, as illustrated in Figure 1. The databases for three samples for various phase loads and *R* = 0.5 have been presented, giving the general damage accumulation of the investigated CFRP material. The graphs in Figure 15 combine three curves, i.e., normalized displacement and rotation, representing the actual value at a given time divided by the initial value, and a cumulative counts curve, representing the cumulative number of acoustic events collected by the piezoelectric sensor during fatigue life. Filters in amplitude (<60 dB) and frequency (<120 kHz) were set to deliver the cumulative curves used to define the CDS point, which was determined according to the following assumption:

- The characteristic damage state (CDS) was assumed to represent fatigue life at the end of the first significant gradient of cumulative counts [58].

This region (CDS) can be associated with a particular failure mechanism, i.e., matrix cracking. Analyzing the curves presented in Figure 15, a correlation between displacement/rotation and the cumulative curve can be highlighted (in all cases, the linear stable phase is reached after the CDS point). CDS is assumed where the significant count gradient ends; it correlates with stabilizing normalized displacement and rotation. The rotation and displacement curves are linear in the next stage, progressing gradually, indicating a stable development of damage. No sudden change in stiffness curves is observed for in-phase and 90° out-of-phase load in most of the life, which suggests that the development of macro damage, such as delamination and large-scale fiber failure, governed the last several hundred cycles (unstable, rapid propagation due to the lack of a constrained layer), causing total loss of integrity of the material. The sudden change in displacement and rotation in a 45° out-of-phase load is observed earlier than in other load cases (around 10% of the total fatigue life), suggesting a longer phase of the macro damage development, as indicated in Figure 15b.

Within the fatigue lifetime, matrix cracking, delamination, and large-scale fiber failure play a significant role on a particular length scale, which causes a reduction in stiffness and final decohesion. Regarding the samples investigated, the primary failure mechanism is matrix cracking and debonding. Based on the macroscale experimental observations and FEA, the damage concentration and fatigue failure were observed within the zig-zag region. The cumulative curve for in-phase load shows a minimal gradient of acoustic events in the material. Rapidly after 70% of the total life, a significant increase in signals is observed; in contrast to in-phase load, a remarkable increase in acquired signals for the 90° out-of-phase load is observed, occurring after 50% of the total fatigue life. The steepest curve gradient is observed for a 45° out-of-phase load, suggesting that damage appears within the first few cycles. The collected acoustic signals indicate that phase shift impacts the appearance of the first damage gradient by shifting its characteristic damage state towards an early stage of the fatigue life. The analysis shows a similarity in the sigmoidal pattern between the cumulative counts curve obtained from the AE data and the general function of damage accumulation described in Ref. [37]. The limitation of this qualitative analysis lies in its inability to identify the failure mechanisms occurring at each stage of the fatigue life.

S-N curves comparison

**Figure 16:**
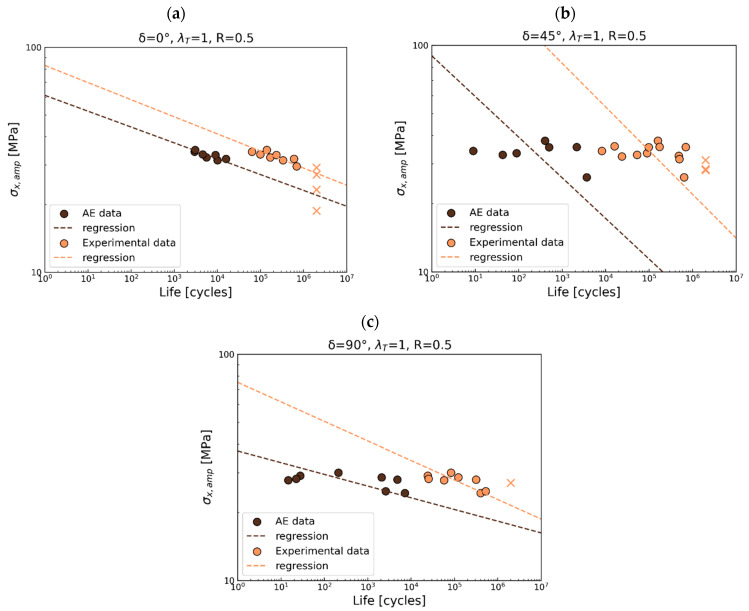
S-N curves comparison for experimental data (total fatigue life) and AE data for R = 0.5 for (**a**) in phase, (**b**) 45° out-of-phase, and (**c**) 90° out-of-phase.

**Figure 17:**
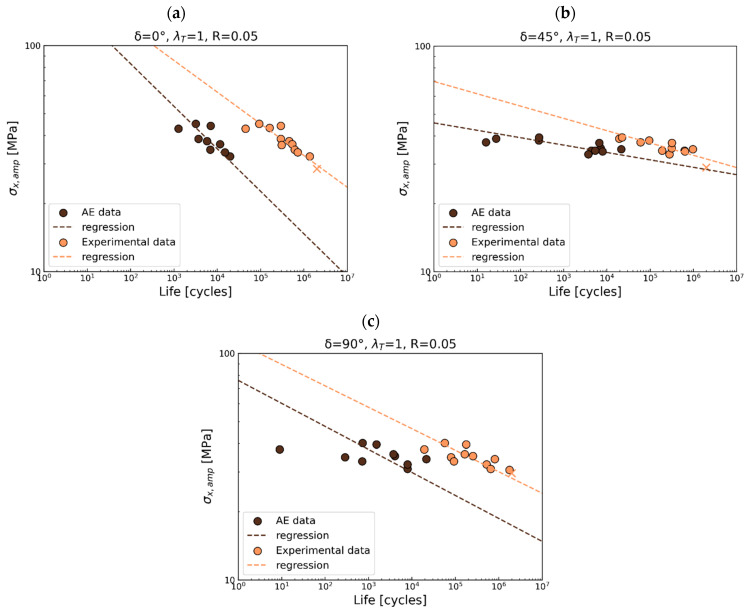
S-N curves comparison for experimental data (total fatigue life) and AE data for R = 0.05 for (**a**) in-phase, (**b**) 45° out-of-phase, and (**c**) 90° out-of-phase.

**Figure 18:**
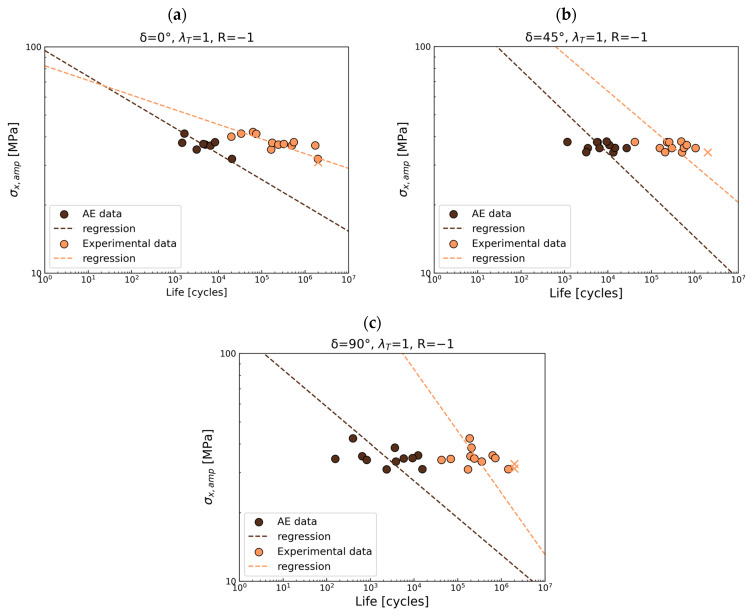
S-N curves comparison for experimental data (total fatigue life) and AE data for R = −1 for (**a**) in-phase, (**b**) 45° out-of-phase, and (**c**) 90° out-of-phase.

As presented in previous sections, the acquired acoustic data were applied to deliver the S-N curves for the first damage gradient recorded by AE. The first failure gradient is associated with the CDS and is correlated with the geometric stress. The obtained S-N curves were compared with the S-N for total life in Figure 16, Figure 17 and Figure 18.

For *R* = 0.5, the first gradient of damage is observed at approximately 4% of the total life under loading in phase, as shown in Figure 16a. A 45° out-of-phase load in Figure 16b shows an initial damage gradient at approximately 0.5%, while a 90° out-of-phase load (Figure 16c) results in a gradient at around 0.5% for short life and around 1% for long life. A difference in the S-N slope is observed only under 90° out-of-phase loads. For the remaining cases, the slopes are parallel. Considering Figure 15b, due to the wide scatter and horizontal nature of the data, statistical analysis is not meaningful.

For *R* = 0.05 (Figure 17a), the presence of the first damage occurs at around 2.5% of the total life for in-phase loading, at 0.5% for short life, and at 2% for long fatigue life under 45° out-of-phase loading (Figure 17b). Variations in the slope of the curve are observed for both in-phase and 45° out-of-phase loads. If the 90° out-of-phase load (Figure 17c) is considered, the first damage gradient is observed at 1% of total fatigue life.

For the reverse load ratio, the first damage gradient is observed at 0.5% for short fatigue life and 1% for long fatigue life under in-phase loading in Figure 18a. For a 45° out-of-phase load in Figure 18b, the damage gradient is observed at 1% of the total life. Finally, the 90° out-of-phase load in Figure 18a,c shows the first damage at 0.5–1% of the total life.

The regression analysis provided indicates that damage appears at approximately 1% of the total life. Variations in the slope of the S-N curves for every *R*-ratio are notable for the 90° out-of-phase load. The delivered S-N curves provide valuable insight into damage-based modeling, as the initial damage gradient can be associated with damage initiation. However, the application of more sophisticated methods, such as machine learning, could help reveal the underlying damage mechanisms during this cycling loading. At this point, only a basic approach has been applied.

## 5. Post-Failure Analysis

**Figure 19:**
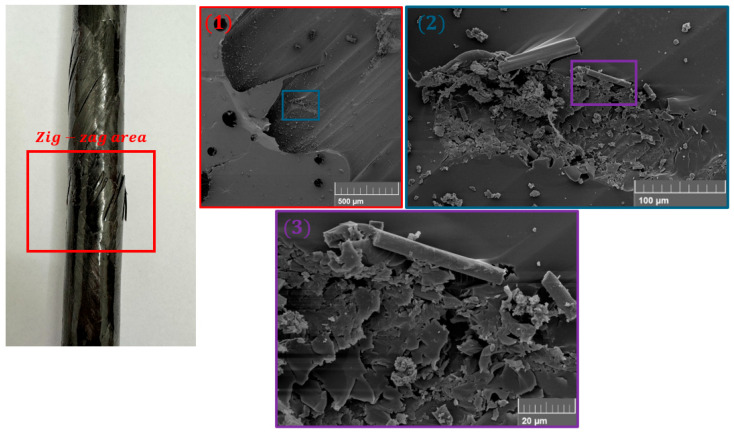
Microscale analysis of the post-failure specimen at the zig-zag area. The images indicate the presence of various types of damage, i.e., external layer porosity, matrix cracking, and fiber breakage.

The investigation of the damage was done by using the SEM and optical microscopy, which allowed for imaging of the fracture area after the specimen failure. In Figure 19, a post-failure analysis done using SEM of a representative specimen is shown. The analysis focuses on the crucial area of the zig-zag where the highest stresses and failure are observed. The figure presents gradually larger magnifications starting with the macroscopic picture of the specimen and then showing consecutive pictures using color-codes, red being the smallest magnification and purple being the largest. One can see different kinds of failure, starting with matrix damage in the (1) picture, through fractured parts of the matrix in (2), to the kinked fibers in (3). The images presented underlined the complex failure behavior of the material, which is a sum of different damage mechanisms.

**Figure 20:**
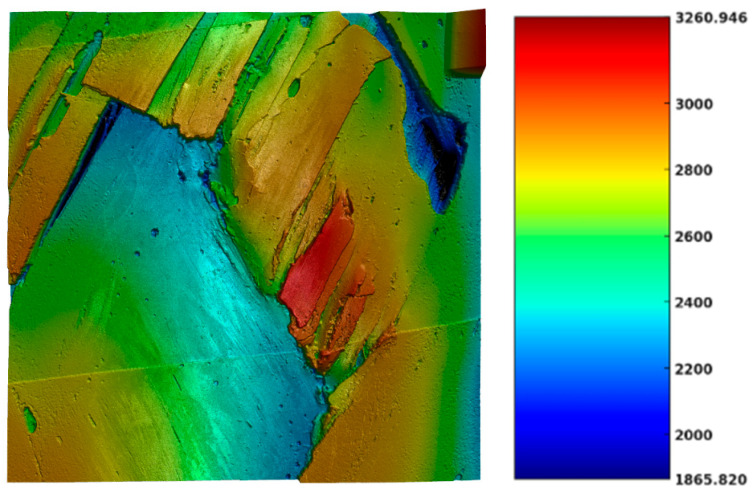
Surface analysis of the zig-zag region in terms of height variations in μm.

Figure 20 presents a 3D topographical surface scan of a filament-wound composite specimen, captured using a confocal microscope KEYENCE VK-X3000 Series. The image displays a damaged region characterized by distinct variations in surface height corresponding to areas of material deformation and failure. The color scale represents surface elevation, where red and orange zones indicate elevated regions, likely caused by delamination-induced surface elevation. Visible cracks and discontinuities, particularly at the boundaries between color transitions, suggest intra-laminar cracking and inter-laminar delamination. The irregular geometry and topography reflect the non-uniform stress distribution typical of the zig-zag regions formed by fiber undulation in filament-wound components.

The damage morphology presented shows that the fracture planes are generally located between the fibers and run parallel to the fiber winding direction. This fracture pattern may be associated with filament-wound structures that contain a limited number of off-axis layers subjected to complex multiaxial loading.

## 6. Conclusions

Phase shift and mean stress effects and their impacts on fatigue characteristics (compared to proportional loading) in a thin-walled CFRP tube (with a 1/1 pattern) are the scientific issues under consideration within the research framework. The following conclusions were drawn based on the multiaxial fatigue experiment enhanced with acoustic emission and numerical stress analysis.

(1) The discrete model incorporated the shear nonlinear material model with progressive damage, allowing for the analysis of the local stress state acting on the material under axial-torsion loads. Due to the inhomogeneity of the stress, a node within a zig-zag area, where $\tau_{12}$ reaches maximum values, was considered. The discrete model includes the effect of fiber undulation, which provides the physical meaning of the filament-wound composites.
(2) Since buckling phenomena can occur during the reverse load ratio, the numerical analysis was run to assess the macroscale stability of the geometry. The numerical analysis based on Riks terms (nonlinear buckling assessment) allowed us to define the Euler force (equal to the maximum value) total of 7.43 kN. When comparing the numerical results with the experimental data, the FEA was found to overestimate the compressive strength. Based on the comparison between the maximum cyclic load applied during the multiaxial tests and the critical static compressive load, a safety margin of 2.86 was calculated. Based on that, it can be assumed that there is no possibility of buckling at the macroscale for multiaxial fatigue performed under *R* = −1. Incorporating the geometrical imperfections into the FE model would improve the accuracy of the results under compressive loading.
(3) The effect of the phase shift has been the objective of this research. The experimental data shows that 90° out-of-phase loads result in shorter fatigue life in comparison to in-phase loads. Due to the large data scatter observed under 45° out-of-phase loading, these results were excluded from the phase-shift analysis, as the effect could not be reliably revealed.
(4) Considering the mean stress effect, there is a noticeable effect on fatigue performance for positive load ratios. The higher the mean stress, the shorter the fatigue life, as the experimental data shows. Due to the large scatter and low correlation of the data under reverse load ratio conditions, a reliable analysis could not be performed.
(5) The damage development analyzed implicitly by displacement and rotational amplitude suggests that the majority of fatigue life covers the stable microdamage development. A more gradual trend is observed for *R* = −1, especially for displacement, which might suggest the impact of additional factors, e.g., local buckling.
(6) The analysis of acoustic signals in terms of the cumulative counts’ function allowed for indirect assessment of the damage accumulation manner in the investigated CFRP material. Based on this analysis, the first gradient of damage was identified as the CDS point and used for delivering the S-N curves. Further investigation would require a deeper analysis of the collected signals to cluster them and associate them with particular failure mechanisms.
(7) Post-failure macro and microscale observations give information about the failure, indicating that in the reference material, the damage affects both the matrix and the fibers. We observe different modes of damage. All of this supports the need to develop more comprehensive approaches for numerical modelling of fatigue behavior in filament-wound pipes.
(8) A deeper damage-based analysis should be performed to reveal the failure mechanisms sequence throughout the fatigue life, as well as the material reference state. While current research provides insight into macroscopic damage accumulation, further investigation of data from acoustic emission combined with observations at the microscale by SEM or CT would help identify the exact nature of damage. This will allow for a better understanding of damage development and failure mechanisms, which is crucial in terms of fatigue behavior modeling.

This study emphasizes the importance of explicitly accounting for mean stress and out-of-phase loading in the design of filament-wound composites. Although the results are preliminary and require deeper damage characteristics, the observed scatter, especially in the 45° out-of-phase case, suggests that more conservative design approaches may be necessary. The present findings provide a foundation for fatigue damage modeling, where life is primarily governed by initiation. Ultimately, these results offer a quantitative basis for defining conservative design factors and supporting their practical implementation.

## Figures and Tables

**Table 1:** Summary of experimental results obtained under external multiaxial loading for unnotched specimens, as reported in the literature.

Authors	Matrix	Fibers	Load Ratios (*R*)	Lay-Up Configuration	Specimen Type
Lee et al. [19]	Epoxy	Carbon woven	0.05	[0/90]_n_	Tubular
Yang et al. [20]	Epoxy	E-Glass	NR	[±45]_4_	Shaft
Gude et al. [21]	Epoxy	E-Glass	0/1/10	-	Tubular
Sun et al. [22]	Epoxy	Glass	10 vertical, 0.1 horizontal	[0/90]_4_ [±45]_4_	Cruciform
Satapathy et al. [23]	Epoxy	Carbon	0.1	[±45]_S_	Tubular
Moncy et al. [24]	Epoxy	Glass UD	0.1	[[0/60/0/−60]_S_/0/60/0/−60]_S_	Cruciform
Lian et al. [25]	Epoxy	Glass	NR	[0]_6_	Tubular
Amijima et al. [26]	Polyester	Glass woven	0	[0/90]_n_	Tubular
Wafa et al. [11]	Polyester	Glass woven	−1	[0/90]_n_ [±45]_n_	Tubular
Fuji et al. [27]	Polyester	Glass woven	0	[0/90]_n_	Tubular
Smith et al. [28]	Polyester	Glass woven	−1	[0/90]_13_ [22.5/112.5]_13_ [±45]_13_	Cruciform
Qi et al. [18]	Epoxy	Glass woven	0/−1	[±35] [±55] [±70]	Tubular
Atcholi et al. [29]	Epoxy	Glass UD	−1	[0]_n_	Bar
Quaresimin et al. [3,14]	Epoxy	Glass woven	0.05/0.1/0.5/−1	[90]_n_ [0/50_2_/0/−50_2_]_S_ [0/60_2_/0/−60_2_]_S_	Tubular
Qiao et al. [30]	Epoxy	Glass UD	0.1	[+45/90/−45/0]_s_ [0/90]_2s_	Notched specimen
Skinner et al. [5]	Epoxy	Carbon woven	0.1/0.3	[0/90]_S_	Cruciform
Weng et al. [10]	Epoxy	Carbon woven	NR	[45/70/0/−70/−45]	Tubular
Zumaquero et al. [6]	Epoxy	Carbon UD	NR	[0]_n_	Cruciform

NR—non-reported.

**Table 2:** Elastic properties of CFRP material reproduced from [42], Elsevier, 2024.

Material	*E*_1_ [MPa]	*E*_2_ [MPa]	*ν*_12_ [-]	*G*_12_ [MPa]
CFRP	128,100	5378	0.345	3132

**Table 3:** Strength properties of CFRP material used in progressive damage modeling reproduced from [55], Elsevier, 2021.

$\mathrm{Transverse}\;\mathrm{Tensile}\;\mathrm{Strength}\;(Y_{t})$	$\mathrm{Longitudinal}\;\mathrm{Compressive}\;\mathrm{Strength}\;(X_{c})$	$\mathrm{Matrix}\;\mathrm{Compressive}\;\mathrm{Strength}\;(Y_{c})$	$\mathrm{Ply}\;\mathrm{Shear}\;\mathrm{Strength}\;(S_{c})$
80 MPa	640 MPa	140 MPa	69 MPa

**Table 4:** The *R^2^* value for CFRP experimental data.

*R* ^2^		Phase Shift		
		0°	45°	90°
R-ratio	−1	0.56	0.03	0.40
	0.05	0.89	0.20	0.66
	0.5	0.70	0.26	0.70

## Data Availability

The original contributions presented in this study are included in the article. Further inquiries can be directed to the corresponding author.

## Nomenclature

$\lambda_{T}$	$\tau_{xy,amp}/\sigma_{x,amp}$(tubular specimen)
α	material non-linearity factor
$\gamma_{12}$	shear strain in the material frame of reference
*δ*	phase shift
*ν_12_*	Poisson’s ratio
$\sigma_{1},\sigma_{2}$	normal stresses calculated in the material frame of reference
$\sigma_{x}$	geometrical normal stress in the laminate frame of reference
$\tau_{12}$	shear stress calculated in the material frame of reference
$\tau_{xy}$	geometrical shear stress
*CDS*	characteristic damage state
*CFRP*	carbon fiber-reinforced polymer
*d*	damage parameter
*DIC*	digital image correlation
$E_{1},\;E_{2},\;G_{12}$	elastic moduli
*FEM/A*	Finite Element Method/Analysis
*FW*	filament winding
*HCF*	high cycle fatigue
*R*	load ratio
*S4R*	4-node, quadrilateral, stress/displacement shell element with reduced integration and a large-strain formulation
*USDFLD*	user-defined field
*UTS*	Ultimate Tensile Strength
*WP*	winding pattern
	*Subscripts and superscripts*
*amp*	amplitude
*n*	non-symmetric
*s*	symmetric
*i*	increment number
*1*, *2*, *3*	material axes
*x*, *y*	laminate axes
